# Mechanism and role of mitophagy in the development of severe infection

**DOI:** 10.1038/s41420-024-01844-4

**Published:** 2024-02-19

**Authors:** Lixiu Ma, Tianyu Han, Yi-an Zhan

**Affiliations:** 1 Department of Respiratory and Critical Care Medicine, the 1st Affiliated Hospital, Jiangxi Medical College, Nanchang University, Nanchang, 330006 Jiangxi China; 2 Jiangxi Institute of Respiratory Disease, the 1st Affiliated Hospital, Jiangxi Medical College, Nanchang University, Nanchang, 330006 Jiangxi China

**Keywords:** Infection, Mitophagy

## Abstract

Mitochondria produce adenosine triphosphate and potentially contribute to proinflammatory responses and cell death. Mitophagy, as a conservative phenomenon, scavenges waste mitochondria and their components in the cell. Recent studies suggest that severe infections develop alongside mitochondrial dysfunction and mitophagy abnormalities. Restoring mitophagy protects against excessive inflammation and multiple organ failure in sepsis. Here, we review the normal mitophagy process, its interaction with invading microorganisms and the immune system, and summarize the mechanism of mitophagy dysfunction during severe infection. We highlight critical role of normal mitophagy in preventing severe infection.

## Facts

1. Mitochondrion is a bacteria-like organelle, and regulates cellular metabolism, differentiation and death. Mitophagy is the last resort to maintain mitochondrial health. Dysfunctional mitochondria and insufficient mitophagy lead cellular stress and severe infection.
2. Intracellular pathogens and inflammatory pathway participate in inhibiting complete mitophagy during severe infection. The exact mechanisms need more study.
3. No selective drug to inhibit mitophagy without influencing other autophagy for research.

## Open questions

1. Do intracellular pathogens block degradation of mitophagosome in the way like disturbing xenophagy?
2. Is mitophagy dysfunction crucial for mild to severe infections?
3. How to balance anti-inflammation (mitophagy) and pro-inflammation (mitochondria) in the infectious diseases?
4. Can extracellular vesicles containing mitochondrial components in the circulation be used as a biomarker of mitophagy condition in the severe infections?

## Introduction

Sepsis is a severe infection with one or more organ failures caused by an imbalance between the host defense and invading pathogens [1]. Early hyperinflammation and subsequent immunosuppression are its defining features, and it occasionally deteriorates with aberrant coagulation, hemodynamic disorder, and microcirculatory disorder. Despite recent advances in medicine, in 2017, sepsis accounted for 11 million (10.1–12.0) deaths, or 19.7% (18.2–21.4) of all deaths worldwide [2]. Sepsis is an uncontrolled stage of infection, and avoiding progressing into sepsis is more important than treating it. Therefore, to concentrate on the pathophysiological mechanisms of pre- and post- uncontrolled infection, we substituted “severe infection” for “sepsis”.

Mitochondria supply a large portion of ATP—cellular energy currency, through its electron transport chain [3]. And reactive oxygen species (ROS) is produced as one of its by-products. Mitochondria contain massive amounts of danger-associated molecular patterns (DAMPs) shared with microorganisms and are potential hazards for cells. New data further support the endosymbiont hypothesis that mitochondria originate from ancient alphaproteobacterial [4]. So, injured mitochondria are like intracellular “pathogens”, causing remote inflammation from infected area during severe infection. Injured mitochondria produce less ATP, and mitochondrial membrane leakage causes proapoptotic molecules, such as ROS, mtDNA, and cytochrome *C*, to enter the cytoplasm [5, 6]. Increased mtDNA in the peripheral blood circulation is associated with poor prognosis of sepsis and coronavirus disease 2019 [7, 8]. Therefore, maintaining mitochondrial homeostasis is critical for preventing and treating severe infection [3].

Autophagy is an evolutionarily conserved self-protection mechanism in cells. Mitophagy is a type of selective autophagy that eliminates labeled mitochondria or their components to keep intracellular homeostasis. Due to mitochondria as the main energy supplier, mitophagy also modulates cellular metabolism. Promoting mitophagy plays protective roles in neuromuscular disease, cancer, and ischemic diseases [9]. In recent years, an increasing number of studies have indicated the intricate association between infection and mitophagy. Therefore, we review the new researches about the mechanism of the mitophagy pathway and its involvement in severe infections.

## Molecular pathways of mitophagy

**Fig. 1:**
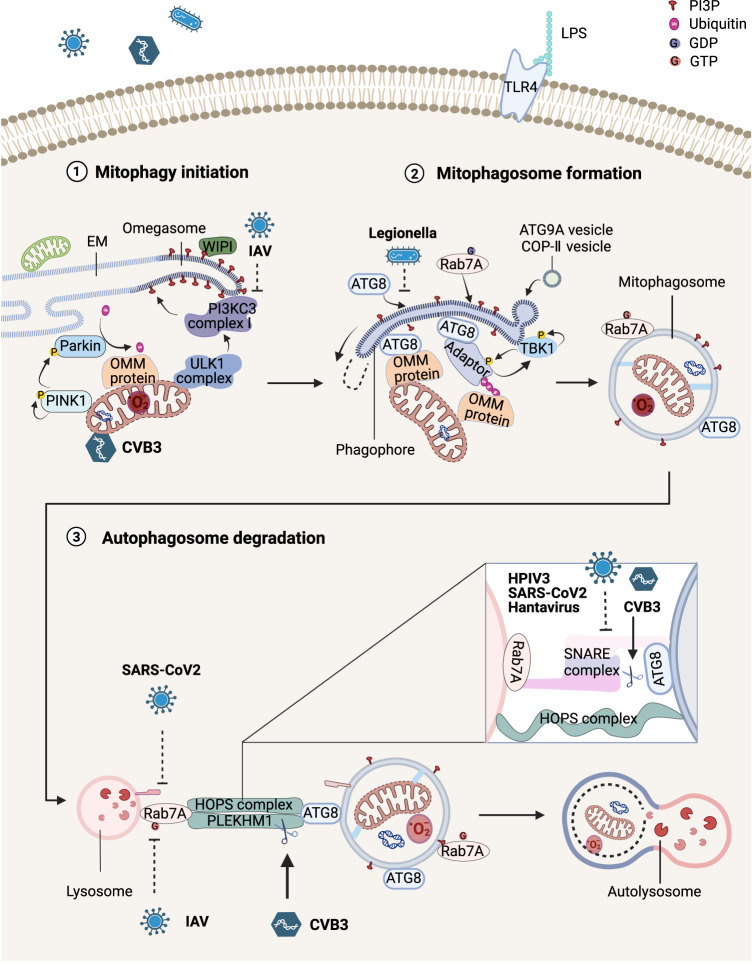
Mechanism of macromitophagy. After cells suffer endogenous and exogenous stimulators, mitophagy is initiated to clear injured mitochondria by recruiting PINK1-Parkin to ubiquitinate mitochondria. The ULK1 complex and PI3KC3 complex I synthesize robust PI3P at the omegasome. Autophagy adapters or receptors bind ATG8 to anchor mitochondria to the phagophore. The double membrane of the phagophore lengthens and enwraps mitochondria. Mitophagosomes fuse with lysosomes mediated by the tethering proteins (such as HOPS complex and PLEKHM1) and SNAREs. Finally, lysosomal enzymes degrade the inner membrane and contents of the mitophagosome. Various microorganisms, such as CVB3, IAV, *Legionella*, HPIV3, SARS-CoV-2, and Hantavirus, disturb the process of mitophagy, resulting in mitochondria injury and autophagosome accumulation. ATG autophagy-related, COP-II coat protein complex-II, CVB3 coxsackievirus B3, ER endoplasmic reticulum, HOPS homotypic fusion and protein sorting, HPIV3 human parainfluenza virus 3, IAV influenza A virus, LPS lipopolysaccharides, PI3P phosphatidylinositol-3-phosphate, PINK1 PTEN-induced putative kinase 1, PLEKHM1 pleckstrin homology domain-containing protein family member 1, RAB7A RAS oncogene family 7A, SARS-CoV-2 severe acute respiratory syndrome coronavirus 2, SNARE soluble N-ethylmaleimide-sensitive factor attachment protein receptor, TBK1 TANK binding kinase 1, TLR toll-like receptor, ULK1 unc-51 like autophagy activating kinase 1, WIPI WD repeat domain phosphoinositide interacting.

According to the initiation mechanism, autophagy is classified as a selective or nonselective pathway. The former includes macromitophagy and micromitophagy. Macromitophagy is a type of classical mitophagy, that is to say, similar to other selective autophagy types (aggrephagy, xenophagy, and lipophagy) [10]. The macromitophagy procedure is essentially divided into three parts: 1) As injured mitochondria are monitored, autophagosome precursors and phosphatidylinositol-3-phosphate (PI3P, or PtdIns3P)-labeled omegasomes, are formed. 2) A double-membrane phagophore (also known as isolation membrane) extends and enwraps damaged mitochondria. Mitophagosomes form as soon as the phagophore is enclosed. 3) Mitophagosomes blend into lysosomes, and mitochondria are dissolved (Fig. 1). The mechanism of micromitophagy is not entirely clear and unspecified mitophagy is macromitophagy in this review.

### Macromitophagy

#### Initiation of macromitophagy

The key signal for macromitophagy is ubiquitinated proteins (Ub-proteins) on the outer mitochondrial membrane (OMM), and the PTEN-induced putative kinase 1 (PINK1)-Parkin pathway primarily contributes to the occurrence of macromitophagy [11]. Under normal conditions, the OMM has a small amount of ubiquitin, and the translocator of the outer membrane-translocator of the inner membrane complex constantly transports PINK1 into mitochondria to maintain its low expression. Once mitochondria are damaged with collapsed mitochondrial membrane potential, the mitochondrial import mechanism becomes clogged, causing PINK1 to accumulate on the OMM and become active by autophosphorylation. Then, PINK1 phosphorylates ubiquitin (Ub) on the OMM and recruits Parkin (E3-Ub ligase) to amplify mitochondrial ubiquitination [12]. Ubiquitin-specific peptidases (USPs), such as USP15, USP 30, and USP33, negatively regulate macromitophagy by hydrolyzing the Ub chains of mitochondrial surface proteins [13–15]. And USP30 also has an auto-inhibiting activity to finely regulate mitophagy [16].

Some phospho-ubiquitin on the OMM combines with autophagy adapters for the next step, and other Ub proteins are destroyed by the proteasome to cause OMM rupture [17]. Mitofusin 2 (MFN2), a GTPase for mitochondrial fusion, also links two membranes of mitochondria and the endoplasmic reticulum (ER), a site called the mitochondria-ER associated membrane (MAM) [18], which is a site of initiating mitophagy. MFN2 dissipation by proteasome promotes mitochondrial fission and isolates mitochondria from the ER and exposes MAM proteins to promote macromitophagy initiation [19]. *Mfn2* deficiency changes the shape of mitochondria. However, it surprisingly suppresses mitophagy and lipogenesis in the alveolar type II epithelial cells. These results also indicate that the physiological processes are mutually intertwined [20].

The UNC-51-like autophagy-activating kinase 1 (ULK1) complex is superior to cell autophagy and a major regulating target of autophagy activity [21–23]. It modulates autophagy by phosphorylating autophagy-related proteins, including ATG13, ATG14L, BECN1, VPS15, VPS34, ATG9A, AMBRA1 and FUN14 domain containing protein 1 (FUNDC1) [24, 25]. Under stress, ULK1 is located at the OMM and promotes the recruitment of downstream autophagic machinery. ULK1 complex (ULK1/2, FIP200, ATG13, and ATG101) is composed at this time to initiate mitophagy. Its formation requires the assistance of autophagy adapters. Among the diverse autophagy adapters participating in macromitophagy, nuclear dot protein 52 (NDP52) and optineurin (OPTN) are the primary proteins that first reside at the OMM and recruit the upstream molecule ULK1 [22]. Adapter proteins mainly recognize microtubule-associated protein 1 light chain 3 (MAP1LC3/LC3) on the phagophore, which is the mammalian homolog of yeast ATG8, through its LC3-interacting region (LIR) to stabilize the phagophore on the mitochondrial surface [11]. Zhou et al. found a sequential relationship between these two roles of adapters. They suggested that autophagy adapters competitively bind FIP200 (scaffold of the ULK1 complex) prior to ATG8 to form the ULK1 complex for initiating autophagy [25, 26]. TNIP1 (TNFAIP3-interacting protein 1), a negative regulator of NF-κB activation, inhibited mitophagy activation via competitively binding FIP200 and TAX1BP1 with autophagy adapters [23].

The ULK1 complex helps to construct class III phosphatidylinositol 3-kinase complex I (PI3KC3-C1) on the omegasome [27], which is another critical initiation complex for autophagy. PI3KC3-C1, consisting of Bcl-2 interacting protein 1 (BECN1, also named BECN1), ATG14L, lipid kinase vacuolar protein sorting 34 (VPS34), and protein kinase VPS15, synthesizes abundant PI3P on the OMM and MAM. The PI3P-positive area of the ER, known as the omegasome, is the origin of the phagophore [28]. PI3P then recruits downstream effector proteins such as double FYVE-domain containing protein 1, WD repeats domain phosphoinositide interacting (WIPI) protein, and soluble N-ethylmaleimide-sensitive factor attachment protein receptors (SNAREs) [29, 30].

Some mitochondrial proteins directly combine with ATG8 via their own LIR motif to participate in macromitophagy, which belong to autophagy receptors (summarized in Table 1). They also independently recruit other effectors to initiate mitophagy [31]. Hypoxia and ischemia cause mitochondrial dysfunction and increase mitophagic flux, in which FUNDC1 is the main autophagy receptor [32, 33]. Under oxidative stress, the tyrosine kinase Src becomes inactive to phosphorylate FUNDC1 and dephosphorylated FUNDC1 gains the function of binding ATG8 [34]. Furthermore, the transcription of autophagy receptors BCL-2 interacting protein 3 like (NIX/BNIP3L) and BCL-2 interacting protein 3 (BNIP3) are increased under hypoxia stress, which belong to the B-cell lymphoma 2 (BCL) family BH3-only proteins that drive macromitophagy and death [35, 36]. Autophagy receptors mediating macromitophagy in severe infection are summarized in Table 1.

**Table 1:** Autophagy receptors participate in macromitophagy in severe infections.

Name	Subcellular location	Molecular interaction	Special pathophysiological effects
Bcl2 interacting protein 3 like (NIX/BNIP3L) [35, 76, 187–189]	OMM	Binding ATG8; Recruiting Parkin to mitochondria	Participating in reticulocyte maturation and macrophage activation; Protecting I/R injury ischemia–reperfusion in the cerebrum; SARS-CoV-2 ORF10 induces macromitophagy by combining with NIX; Promoting HHV-8 replication
Bcl2 interacting protein 3 (BNIP3) [118, 190–192]	OMM	Binding ATG8; Inhibiting degradation of PINK1; Recruiting DRP1	Coordinating the process of apoptosis and macromitophagy against cell stress; Alleviating sepsis kidney injury; Promoting generation and survival of memory immunocytes
Bcl-2 like 13 (BCL2L13) [193–195]	OMM	Binding ATG8; Recruiting DRP1 and ULK1	Belonging to BH3-only proteins to coordinate the role of apoptosis and macromitophagy against cell stress
Mcl-1 [181, 196]	OMM	Binding ATG8; Binding BAX/BAK	Inhibiting BAX/BAK induced apoptosis; Promoting Ub-independent mitophagy
FUN14 domain-containing protein 1 (FUNDC1) [32, 197–200]	OMM/MAM	Dephosphorylated FUNDC1 binds ATG8; Recruiting ULK1 and DRP1	Inhibiting IL-1β secretion in macrophage and alleviating septic lung injury and cardiomyopathy; Regulating mitochondrial dynamics
Activating molecule in Beclin1-regulated autophagy 1 (AMBRA1) [201–203]	From cytoplasm to OMM	Binding ATG8 or ATAD3A; Activating PIK3C3 complex 1 by binding BECN1	Compensating for the deficient PINK1/Parkin-dependent mitophagy in Parkinson disease; Protecting neurons from hypoxia; Blocking PINK1 import into mitochondria
Prohibitin2 (PHB2) [105, 204, 205]	IMM	Binding ATG8; Stabilizing PINK1 on the OMM	Alleviating septic kidney injury; Facilitating macrophage to scavenge *P. aeruginosa* and mROS
Cardiolipin [110]	From IMM to OMM	Binding ATG8	Promoting mitochondrial apoptosis or macromitophagy in the severe infection

#### Formation of mitophagosomes

On the base of the omegasomes, the phagophores gradually extend and eventually close up. Multiple budding sites are simultaneously built on the mitochondrial surface cargos to exceed mitophagosome formation [37]. This process acquires various lipids and membranes supplied from the endomembrane system [38]. At same time, free ATG8 covalently links phagophore lipids (Phosphatidylethanolamine) catalyzed by ATG7(E1 ligase), ATG3 (E2 ligase), and the ATG16L1-ATG12-ATG5 complex (E3 ligase). Lipdated ATG8 fixes the phagophore to the mitochondrial surface.

Intracellular vesicles, which originate from the EM, Golgi bodies, and plasma membrane, are one of the most important lipid sources for phagophores [39]. Multiple membrane dynamics regulators, primarily SNARE proteins, tethering proteins, and Rab GTPases, precisely regulate membrane formation, transport, and destruction, in which Rab7A is the core element [40]. Quick dynamics and interaction of these molecules are necessary for efficient autophagy.

The activity of Rab GTPases is regulated by the activator (guanine nucleotide exchange factor) and the inhibitor GTPase-activating protein. Guanine nucleotide exchange factor1 early moves to the OMM by binding Ub and recruits Rab5 to facilitate Rab7A translocation and activation [41]. Rab7A further recruits SNAREs and tethering proteins to regulate movement and fusion of intracellular vesicles [41]. TBC1D5, MON1-CCZ1 complex, and C5orf51 shift Rab7A from lysosome to OMM/phagophores/autophagosome in favor of contineous Rab7A circulation [42, 43]. ATG9A vesicles, which are necessary for early phagophore generation, move to the macromitophagy site or phagophore guided by Rab7A and its effectors (such as the retromer complex and sorting nexin 18) [38, 42, 44, 45], and SNX4 participates in ATG9A recycling from autolysosomes [46]. The adapter protein OPTN binds ATG9A to assist ATG9A vesicle capture [47]. COP-II vesicles also participate in autophagosome formation [48].

Aside from transporting lipids over long distances, membrane sources can be directly assimilated near the phagophore. In the early stage, the lipid transfer protein ATG2A builds a bridge for the membrane of the ER flowing to the phagophore, with one end touching the ER and the other end binding the WIPI protein on the border of the phagophore [49, 50]. In addition, lipid droplets near the phagophore are decomposed by lipase PNPLA5 to supply ingredients for phospholipid biogenesis [51].

The phagophore gradually encompasses the mitochondria, and the double membrane of the mitophagosome is enclosed once its blind ends fuse.

#### Degradation of mitophagosomes

Complete macromitophagy ends with mitophagosomes degradation. Under physiological conditions, ATG8 and PI3P recruit regulators of membrane dynamics to the surface of mitophagosomes/lysosomes. Two types of vesicles move toward each other along microtubules mediated by Rab7A effectors [52–54].

Then, the tethering proteins, homotypic fusion and protein sorting (HOPS) complex and Pleckstrin homology domain-containing protein family member 1 (PLEKHM1), bind them with their long and stiff structure at the same time [55]. After the HOPS complex softens, two vesicles get near each other, and short SNARE proteins on each surface incorporate SNAP29 into the heterotrimeric complex (YKT6-SNAP29-STX17 or STX17-SNAP29-VAMP7/8) to facilitate the touch and fusion of their outer membranes [56, 57]. Then, lysosomal enzymes flow into the intermembrane space and dissolve the inner membrane of autophagosome to turn it into one vesicle.

Mitophagosomes can also fuse with cell membranes and get out of the cell to maintain mitochondrial homeostasis, a process known as secretory autophagy, when the degradation of mitophagosomes is blocked or insufficient to clean injured mitochondria [58, 59].

### Mechanism of micromitophagy

Micromitophagy is a highly selective method of eliminating harmful mitochondrial components to avoid costly macromitophagy, which runs steadily in the cells with high metabolic demand. The micromitophagy also compensates for deficient macromitophagy to cope with oxidative stress [60, 61]. Micromitophagy generates two kinds of mitochondrial-derived vesicles (MDVs) in a completely distinct way: pyruvate dehydrogenase-positive MDV and TOMM20-positive MDV. The former buds from the OMM, which is regulated by PINK1 and Parkin, and fuses with lysosome like the mitophagosome pathway [62]. The latter is controlled by MIRO1 (microtubule-associated motor proteins mitochondrial Rho GTPase 1) and DRP1, which traffics to multivesicular bodies and lysosomes in the absence of the SNARE complex. Another study discovered that Rab9 and sorting nexin 9 mediate MDV budding under oxidative stress [63, 64]. Furthermore, injured mtDNA and mitochondrial proteins can only be removed from mitochondria and broken down in the lysosome [65, 66].

#### Mitophagy and severe infection

In sepsis, mitochondrial dysfunction is obvious and associated with disease progression. Macromitophagy/mitophagy level increases in the early stage but fails to resolve serious mitochondrial injury, and it oddly decreases in the late stage, resulting damaged mitochondria accumulating in the cells [67]. According to RNA-seq of the whole blood, patients in ICU have lower mitophagy levels compared to patients in emergence room, and patients with lower mitophagy have higher SOFA scores [68]. Defective autophagy is also associated with lymphopenia in COVID-19 [69]. Thus, we discuss the relationship between severe infection and mitophagy in detail below.

### The connection between mitophagy and microorganisms

#### Pathogens induce mitophagy to suppress host defense

RIG-I-like receptors (RLRs) with mitochondrial antiviral signaling protein (MAVS) recognize double-stranded RNA of pathogens in the cytoplasm to induce the production of interferon (IFN) and other proinflammatory factors [70, 71]. However, matrix protein of human parainfluenza virus 3 (HPIV3), PB1-F2 protein of influenza A virus (IAV) and glycoprotein of hantavirus, like an autophagy adapter, link with ATG8 and mitochondrial Tu translation elongation factor (TUFM) to activate Ub-independent mitophagy for blocking MAVS signaling [72–74]. The nucleoprotein of IAV also binds MAVS and LIR-containing TOLLIP (toll interacting protein) to promote mitophagy [75]. Another viral protein, severe acute respiratory syndrome coronavirus 2 (SARS-CoV-2) open reading frame 10 (ORF10), induces mitophagy by building a bridge between NIX and LC3B [76] (Fig. 1). It is probably a common way for pathogens to suppresses innate immune responses and causes persistent infection via directly or indirectly promoting mitophagy.

#### Pathogens escape from elimination in host cells by hijacking autophagy mechanisms

Intracellular microorganisms are also marked with Ub and degraded by the autophagy pathway, named as xenophagy. However, some pathogens can hijack phagosomes/autophagosomes for their own replication and secretion instead of fusing with lysosomes. Major targets disturbed by pathogens are SNARE proteins, RAB7A, and tethering proteins: HPIV3-derived phosphoprotein and hantavirus-derived nucleocapsid protein bind SNAP29 and inhibit its interaction with syntaxin17 [74, 77]; SARS-CoV-2 ORF7a promotes caspase 3 to hydrolyze SNAP29 [78, 79]; SARS-CoV-2 ORF3a binds VPS39, a subunit of the HOPS complex, to obstruct the HOPS complex from combining with Rab7A on the lysosome surface [80]; IAV M2 enhances the depressant effect of TBC1D5 on Rab7A [78]. A recent article reviewed more details of coronaviruses disturbing the autophagy process [81]. Coxsackievirus B3, which causes severe myocarditis and systemic infection, directly localizes to mitochondria and induces DRP1-mediated mitochondrial fission [82]. And its proteinase 3C causes incomplete mitophagy in the host cell via cleaving PLEKHM1 and SNAP29 [83]. Several bacteria also block autophagy mechanisms to lead to acute or chronic infection, such as *Legionella pneumophila* and *Salmonella* [84, 85] (Fig. 1).

Due to the similar degrading progress of different autophagosomes, it is reasonable that intracellular pathogens mentioned above also block mitophagosome degradation, resulting in severe inflammation and host cell death.

### Mitophagy regulates immune disorders in severe infection

Characterized by an intense inflammatory response or cytokine storm, sepsis in the late stage often turns to immunosuppression, making the host susceptible to secondary infection and increasing mortality. Pathogens, neutrophil extracellular traps, platelets, and endothelial cells all work together to cause hyperinflammation, but immunosuppression is related to long-term infection and increased immune cell death, functional fatigue, and anti-inflammatory milieu. Based on these mechanisms, two major ways to treat sepsis are inhibiting early excessive inflammation and ameliorating late immunosuppression [86, 87].

Most leukocytes activation is regulated by metabolism switch and mitochondrial ROS (mROS) level. Glycolysis promotes proinflammatory subtypes differentiation, and oxidative phosphorylation and fatty acid metabolism are required by differentiation of negatively regulating subtypes. However, metabolic dysregulation and mitochondria injury is outstanding in the immunocytes from severe infection and leads to their dysfunction [88]. Mammalian target of rapamycin (mTOR) and AMP kinase (AMPK) are a pair of cellular energy sensors that have opposite effects on cell metabolism and immune cell differentiation. mTOR simultaneously inhibits suppresses ULK1 phosphorylation and autophagy-related protein expression [89–91], which are antagonized by active AMPK. Thus, mitochondria have close connection with immune disorders in severe infection and targeting mitophagy or its regulators is beneficial for immunometabolism back to normal.

#### Mitophagy reduces hyperinflammation in the sepsis

Mitochondria and their components have a risk of causing uncontrolled inflammation while activating innate immune responses to resist infection. First, mitochondria supply an effective platform for the NLR family pyrin domain containing 3 (NLRP3) inflammasome activation: MAVS and cardiolipin on the OMM directly bind NLRP3 and pro-caspase 1, and mitochondrial E3 ligase MARCH5 ubiquitinates NLRP3 on K324 and K430 to facilitate NLRP3 oligomerization [92]. VDAC oligomers, mROS, and mtDNA assist in assembling NLRP3 inflammasome and activating caspase 1 [93]. Caspase 1 not only mediates the release of inflammatory factors and pyroptosis but also worsens mitochondrial injury and dampens mitophagy by degrading PINK1, Parkin, and ATG8 [94]. Increased mROS and oxidative mtDNA are also associated with PANoptosome assembly and PANoptosis [95]. Second, cytoplasmic mtDNA is recognized by cyclic guanosine monophosphate–adenosine monophosphate synthase, which activates stimulator of IFN genes (STING) to promote proinflammatory reactions. Third, extracellular mitochondrial components, free or encompassed in the vesicles, can cause remote and systematic inflammation. Macrophages and other cells are activated after engulfing these vesicles [96, 97]. MDVs are involved in mitochondrial antigen presentation to induce autoimmune reactions and may cause immunological injury during severe infection [63, 98]. Lysosome disposes MDVs and presents mitochondrial components by MHC class I molecules on the cell membrane. Then marked cell is recognized and killed by CD8^+^ T cells.

B cells and dendritic cells with impaired autophagy (ATG5 deletion) develop a sterile sepsis-like inflammatory condition, and accumulative cardiolipin accounts for this condition [99]. Thus, it is evident that promoting mitophagy, which removes relative incentives to block pro-inflammatory pathway, aids in inflammation control and cells survival [100]. Endosomal protein APPL1, which shuttles among several organelles to regulate cell proliferation and death, plays a feedback mechanism to inhibit NLRP3 inflammasome through interacting with Rab5 to promote mitophagosome degradation [101]. Sestrin 2, a stress-inducible protective protein, suppresses macrophages’ NLRP3 inflammasome activation by upregulating ULK1 expression and recruiting autophagy adapter p62 to induce mitophagy [102, 103]. The M2 protein of IAV is located at the OMM and then enhances MAVS assembly, resulting in a dramatic antiviral reaction and host cell death, which is alleviated by mitophagy [104]. Gram-negative bacterium *Pseudomonas aeruginosa* causes severe respiratory infection. Huang et al. found that microRNA-302/367 enhanced Prohibitin2-induced mitophagy to eliminate *P. aeruginosa* and ameliorate oxidative stress in alveolar macrophages [105].

#### Mitophagy alleviates septic immunosuppression

Sepsis in the immunosuppression state presents an increasing apoptotic ratio and functional exhaustion in immunocytes, such as CD4^+^ T cells, CD8^+^ T cells, B cells, Natural Killer cells, and monocytes [106].

**Fig. 2:**
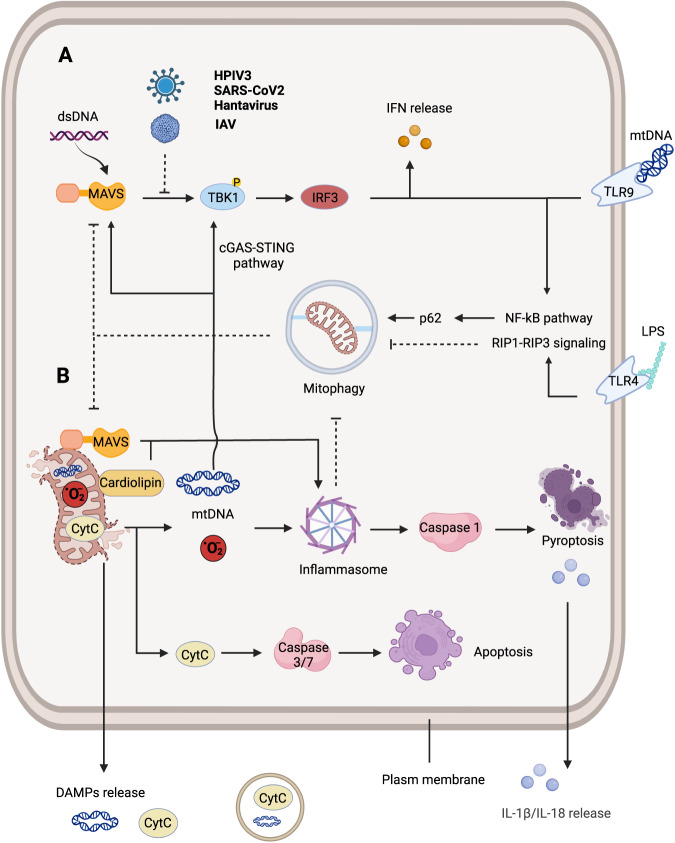
Mitophagy interacts with the inflammatory cascade. **A** Mitochondria contribute to MAVS-induced anti-infective response. mtDNA promotes interferon expression by cGAS-STING pathway. HPIV3, SARS-CoV2 and Hantavirus inhibit the role of MAVS to survive themselves. **B** DAMPs derived from mitochondria induce apoptosis and pyroptosis in host cells by activating caspase 1/3/7. Mitophagy blocks inflammatory pathway via scavenging mitochondria-derived DAMPs. TLR-NF-κB signaling promotes p62-dependent mitophagy to inhibit mitochondria-dependent inflammation and cell death. TLRs also activate RIP1-RIP3 signaling to induce incomplete mitophagy and necroptosis. cGAS-STING cyclic guanosine monophosphate–adenosine monophosphate synthase and stimulator of IFN genes. IRF interferon regulatory factor, MAVS mitochondrial antiviral signaling protein, mtDNA mitochondrial DNA, dsDNA: double strand DNA, Cyt *c* Cytochrome *c*, DAMPs danger-associated molecular patterns, RIP receptor interacting protein.

Mitochondria play critical roles in cell survival and apoptosis. Mitochondrial DAMPs get a chance to enter the cytoplasm after pro-apoptotic BCL-2 proteins BAX-BAK induce mitochondrial outer membrane permeabilization and cyclophilin D triggers mitochondrial permeabilization transition in the inner mitochondrial membrane (IMM). Cytochrome *c* and mtDNA activate caspase-3/7 to execute lethal or sublethal apoptosis [6, 107] (Fig. 2). Interestingly, the mitophagy is simultaneously initiated with BAX-BAK activation to block mtDNA-dependent inflammation [108]. Cardiolipins in the IMM translocate to the OMM and recruit caspase-8, which activates caspase-3/7 [109], which also promotes mitophagy as an autophagy receptor [110]. Some BCL-2 proteins are also mitophagy receptors, such as BECN1, NIX, BNIP3, and BCL213L (Table 1). Therefore, cells under proapoptotic stress will survive and decrease unnecessary inflammation if mitophagy is timely initiated.

After the immune system goes all out to fight against invasive pathogens, effector cells accumulate damaged mitochondria and other organelles, which may go into an exhausted precursor population. Consistent mitochondrial dysfunction and redox stress cause T cells into terminal exhaustion [111]. Autophagic flow decreased in T cells after cecal ligation surgery (animal model of sepsis) for 24 h, and deficient autophagy or mitophagy via knocking out key autophagic molecules causes abnormal differentiation and apoptosis in T cells, natural killer cells [112–114]. Mitophagy survives effector CD4^+^ T cells by inhibiting the production of mTOR pathway-dependent mROS [90]. The role of dendritic cells (DCs) is also impaired in sepsis and the state of DCs positively correlated with the level of PINK1-dependent mitophagy [115, 116]. The B cells in the germinal center exhibit a highest mitophagy rate [117]. Furthermore, enough autophagy, including mitophagy, is critical for formation of immune memory by promoting oxidative phosphorylation reversion and decreasing ROS level and effector immunocytes apoptosis [118–120]. This is meaningful for reducing risk of reinfection after severe infection and increasing long-term survival rate.

#### Mitophagy participates in the self-regulation of inflammatory pathways

Not only does the NLRP3 inflammasome inhibit mitophagy to rapidly amplify anti-infective reactions, but some inflammatory pathways also facilitate mitophagy to ensure appropriate host defense. Toll-like receptors (TLRs) bind their ligands, such as LPS and mtDNA, and transduce signals inside to activate the NF-κB pathway [121]. Along this pathway, NF-κB increases the expression of p62, which recognizes ubiquitin-tagged mitochondria, to promote mitophagy-mediated suppression of the NLRP3 inflammasome [122] (Fig. 2). MAVS acts as an autophagy receptor, and TUFM binds ATG5-ATG12 to initiate mitophagy to degrade MAVS, which is a self-regulation of the antiviral response [123, 124].

TANK binding kinase 1 (TBK1) is a critical kinase downstream of the TLR3/7/8/9, RLR, and GAS-STING pathway, and it also positively mediates autophagy process [125, 126]. On the one hand, active TBK1 phosphorylates the transcription factor of interferon regulatory factor-3, which induces the gene expression of type I IFN and additional chemokines. On the other hand, TBK1 is recruited to the OMM by NDP52 and OPTN. After its autophosphorylation and activation, TBK1 phosphorylates binding sites between autophagy adapters (NDP52, OPTN, p62, and Tax1 binding protein 1) and ATG8/Ub to enhance their affinity. This positive feedback loop between TBK1 and NDP52/OPTN accelerates the autophagy process [127, 128]. ATG8 phosphorylation by TBK1 steadily binds on isolation membrane [129]. TBK1 also facilitates NDP52 recruiting ULK1 to the OMM [22]. Additionally, Rab7A phosphorylated by TBK1 promotes ATG9A vesicle recruitment for autophagosome formation [130]. Thus, TBK1 is a self-limiter for inflammatory response. Furthermore, another review gives an opinion that two pathways of TBK1 collectively contribute to kill intracellular pathogens. TBK1-dependent autophagy/xenophagy activation plays major roles in antibacterial action and IFN response is an epiphenomenon [131].

Severe infection causes a breakdown of the equilibrium between mitophagy and inflammation, and exact mechanisms remain unknown. Intracellular pathogens and alteration of mitophagy-associated gene expression may contribute to this unbalance. In the last two years, researchers found that the necroptosis pathway promoted incomplete mitophagy and increased the release of mitochondrial components-containing exosomes, which was caused by dysregulated binding of SNARE proteins on mitophagosomes and lysosomes [96, 132].

### Mitophagy protects organ function in sepsis

**Fig. 3:**
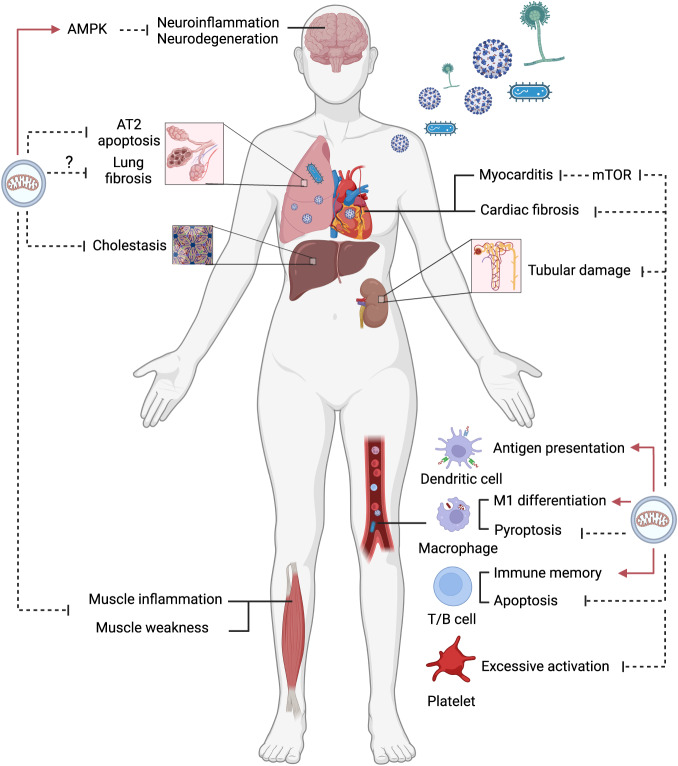
The roles of mitophagy at the tissue and organ levels. Mitophagy inhibits organ dysfunctions, including brain, lung, heart, liver and muscle weakness, and modulates immune cell differentiation. AT2 alveolar type 2, AMPK AMP-activated protein kinase, mTOR mechanistic target of rapamycin.

Sepsis represents a high metabolic state and one or several organ malfunctions, partly caused by mitochondrial injury and insufficient mitophagy (Fig. 3).

#### Lung

Compared with alveolar type (AT) 1, AT2 rapidly coped with mitochondrial damage by boosting mitophagy and mitochondrial biogenesis in the acute lung injury caused by *Staphylococcus aureus*. Thus, AT2 resisted apoptosis more than AT1 [133]. Severe pneumonia usually causes local or diffuse lung fibrosis [134, 135], which is associated with a defect in PINK-Parkin-dependent mitophagy. Thymosin β4 has antioxidant, anti-inflammatory, and antifibrotic effects in LPS-induced lung fibrosis, partly by promoting mitophagy to attenuate oxidative stress in alveolar epithelial cells and fibroblasts [136].

#### Heart

In cardiomyocytes, LPS at a low dose generated an increase in autophagic flow, followed by a decrease at high doses [137]. SARS-CoV-2 infection induces acute myocarditis and cardiac fibrosis, which is associated with its spike-1 protein stimulating the NLRP3 inflammasome and oxidative stress by inhibiting mitophagy [138]. BECN1 acetylation contributes to autophagy suppression, and melatonin protects the septic heart by enhancing sirtuin1-mediated BECN1 deacetylation [139]. Exogenous BECN1 suppresses mTOR signaling and promotes mitophagy to attenuate sepsis-associated myocarditis [137]. Periplaneta americana extracts regulate LPS-induced cardiomyocyte injury via PINK1-Parkin-dependent mitophagy [140].

#### Kidney

Renal tubular epithelial cells are also sensitive to poor oxygen and nutrients. In septic shock, the kidney is compromised with altered renal perfusion (hypovolaemia and high central venous pressure) and immunological response. NLRP3 inflammasome activation impairs mitophagy in septic renal tubule cells [141]. Insulin-like growth factor-binding protein 7 serves as a biomarker for sepsis-associated acute kidney injury (SA-AKI) and contributes to the pathophysiology of SA-AKI via dampening NIX-dependent mitophagy [142]. PINK1-Parkin-OPTN axis and BNIP3-mediated mitophagy also promote tubular cell survival [36, 143]. Recently, Deng and coworkers found that melatonin was increased in SA-AKI and played a protective role in septic renal tubule cells. They verified that melatonin promoted mitophagy and maintained mitochondrial quality by activating sirtuin-3-dependent deacetylation of transcription factor A [144].

#### Other organs

The liver, which handles almost all nutrient and drug metabolism, is easily damaged in sepsis. Blocking autophagy accelerated mitochondrial dysfunction and apoptosis in hepatocytes. Mitophagy defect aggravates hepatocyte apoptosis and mortality of septic mice [67, 145]. Sepsis-induced hepatic injury presents cholestasis and elevation of aminotransferase. Bile acid retention obstructs the fusion of autophagosomes with lysosomes in hepatocytes [146]. Obeticholic acid, a drug used to treat primary biliary cirrhosis, also maintains bile acid homeostasis in septic liver injury via improving autophagic flux [147]. Sepsis-associated encephalopathy (SAE) is characterized by sterile inflammation and long-term cognitive impairment. The expression of the stress-induced protein sestrin 2 increases in septic neurons, which protects against SAE by activating AMPK pathway to promote neuronal autophagy [148]. Natural components fisetin and urolithin A have been shown to alleviate inflammation in microglia and neurodegeneration by activating mitophagy [149, 150]. Endothelial damage is a major reason for microcirculatory dysfunction, microthrombosis, and organ dysfunction. Neutrophil extracellular traps inhibit mitophagy by inducing FUNDC1 phosphorylation to lead to endothelial ferroptosis, which can be reversed by urolithin A [151].

However, skeletal muscles are highly catabolized to supply nutrients for other key organs in critical illness, so septic patients in hospital and after discharge often suffer muscle weakness [152]. The autophagy pathway participates in protein breakdown [153, 154], resulting in loss of muscle mass and strengthening. Leduc-Gaudet et al. found that Parkin overexpression prevented sepsis-induced skeletal muscle atrophy, which was partly explained by improving mitochondrial quality and alleviating muscle inflammation [155]. Therefore, mitochondrial autophagy, in contrast to aggrephagy, may ameliorate sepsis-associated myopathy.

In conclusion, defective mitophagy mainly contributes to septic organ failure, whereas increased mitophagy protects organ function by alleviating oxidative stress and apoptosis in the cells.

### Mitophagy regulates platelet functions

Platelet activation depends on energy and ROS supplied by rich and healthy mitochondria. Thus, inducing mitophagy compromises platelet activation [156, 157]. For example, hypoxic preconditioning for activating mitophagy in the platelet protects against acute ischemia/reperfusion-induced heart injury in mice [158].

Platelets, as a part of innate immune response, interact with neutrophils and endothelial cells and result in immunothrombosis after microorganisms invade [159, 160]. Local thrombi restrict microorganisms to diffuse with sacrificing microcirculation perfusion. Excessive thrombi are associated with severe illnesses, such as acute respiratory distress syndrome induced by SARS-CoV-2 and sepsis [161–163]. Compared with septic survivors, platelets from non-survivors showed a significant decrease in ATP and an increase in mitochondrial permeabilization [164]. Mitochondrial dysfunction promotes phosphatidylserine externalization of plasma membrane, which greatly increases clotting tendency and uncontrolled immunothrombosis [165]. Meanwhile, platelets in the sepsis represented incomplete (mitochondrial) autophagy due to LPS-TLR4 signaling blocking tether protein EPG5 (ectopic P-granules autophagy protein 5 homolog) binding ATG8 [166]. Therefore, mitochondrial dysfunction and excessive thrombosis in the severe infection are probably reversed by promoting mitophagy.

## Discussion and conclusion

Severe infections are a result of multiple pathophysiologic disorders caused by invading microorganisms. Host will suffer high metabolic and mitochondrial damage during anti-infection. Therefore, cells initiate mitophagy to eliminate injured mitochondria and components to keep intracellular homeostasis, or they undergo intensive proinflammatory reactions and mitochondria-related apoptosis. Various regulators in cells lead to inadequate or incomplete mitophagy, partly resulting in uncontrolled inflammation and organ dysfunction. Thus, mitophagy disorder may be both the cause and result of infection progress. And it is a critical factor for worsening conditions to be determined.

The degrading disorder of mitophagosome is more obvious in critical infections, but detailed mechanisms are not clear. Lysosome dysfunction could explain part of this disorder [167, 168]. Gut microbiome critically regulates immunoreaction in autoimmune diseases and other diseases. Feng Li et al. found that intestinal bacteria, especially *Lactobacillus*, could regulate microglia activation in herpes simplex encephalitis via producing nicotinamide n-oxide to induce NAD^+^-dependent mitophagy [169]. Urolithin A, a dietary metabolite of the intestinal microbiota, has strong effect on anti-inflammation and protecting mitochondrial health [170]. Thus, intestinal flora imbalance might participate in mitophagy disorder of severe infections. The negative regulating mechanisms for mitophagy in other diseases, such as VAMP7B and Rab7A disfunction, should be investigated in critical infections [23, 57, 171, 172].

Reinstating proper mitophagy prevents hyperinflammation and organ failure in severe infections and preserves anti-infective ability. However, it is a challenge to find the right time and strength to intervene mitophagy for balancing the function of anti-inflammation (mitophagy) and pro-inflammation (mitochondria). Due to the yet-to-be-determined intervention process, intricate pathophysiological state of severe infection and no excluded confounding factor (mitochondrial injury), it is likely to cause opposite conclusions about the roles of mitophagy in the severe infection. New evaluation methods of the whole mitophagy state should be established to guide us in regulating mitophagy more scientifically. Extracellular vesicles, containing mitochondrial components in circulation, may be used as a biomarker to estimate mitophagy conditions in severe infections [173].

Importantly, increasing mitophagy is not always beneficial due to complicated functions of mitochondria. First, mitochondria participate in direct and indirect ways to kill pathogens and inhibiting mitophagy in the active macrophages is a physiological process to increase phagocytosis ability and secretion of type I IFN. Mitophagy cleans up proinflammatory pathway activators to block anti-infective processes, which is prone to allow infection to spread. Sometimes, mitophagy is hijacked by intracellular pathogens to finish their lifecycle. Thus, promoting mitophagy may do harm to bodies at early stage of infection [116, 174–176]. Second, there is a close relationship between mitophagy and mitochondrial apoptosis and E3 ligase Parkin can be a converter of them [177]. Several pro-apoptotic and anti-apoptotic proteins are also autophagy receptors to initiate mitophagy. Pro-apoptotic BAX-BAK oligomers induced mitophagy to inhibit unwanted inflammation. Thus, they work together to minimize the damage. But there is a potential risk that external mitophagy enhancers undermine this fine coordination. A study published in *Immunity* journal pointed out that decreasing apoptosis of effector immunocytes, which alleviates immunosuppression in the sepsis, potentially promote lung fibrosis after acute lung injury via incresing long-lived macrophages-derived TGF-β1 [178]. Third, excessive and unnecessary mitophagy without corresponding mitochondrial biogenesis also causes inadequate ATP production and induces cell death. Mitophagy-induced cell death is a high-profile method to treat malignant tumors, but it is not a good thing for applying mitophagy inducers into other diseases, such as severe infections [179].

The pathway, flux, and roles of mitophagy are different in different cells, organs, and severe infection stages. Based on its features and potential risks, some basic principles we suggest should be complied for mitophagy inducers applying to non-malignant diseases: 1) High selectivity for injured mitochondria, target cells, and organs. Nanoparticles, such as extracellular vesicles, have good targeting ability with or without artificial modification and can possibly be used as medicine carriers to satisfy precise modulation of mitophagy in severe infections [180]. And according to the theory of autophagy-tethering compounds (ATTECs) [179], some molecules, which are normally inside the mitochondria and exposed to the cytoplasm once mitochondria dysfunction, may be fit as the binding sites for ATTEC, such as cardiolipin and prohibitin 2. 2) Promoting mitophagy without injuring mitochondria, such as UMI-77 and mito-ATTEC. 3) Appropriate dosage and efficacy of inducers to avoid lethal mitophagy [179, 181].

Aside from macromitophagy and micromitophagy, mitochondrial quality control includes mitochondrial dynamics and mitochondrial biogenesis. Tree of them cooperate mutually to maintain mitochondrial homeostasis—mitochondrial fission assists mitophagy in selective elimination of injured parts, and harmful components are diluted after injured mitochondria fuse with healthy ones. Timely biogenesis of mitochondria is needed to maintain the cellular energy supply [182]. It is reported that PGC-1α/NRF1 raised the expression of FUNDC1 to simultaneously promote mitophagy and mitochondrial biogenesis to maintain mitochondrial functions [183]. Thus, mitophagy inducers with the bioactivity of promoting mitochondrial biogenesis potentially recover mitochondrial functions in severe infections better, such as resveratrol, melatonin, urolithin A and berberine [5, 32, 184–186].

**Fig. 4:**
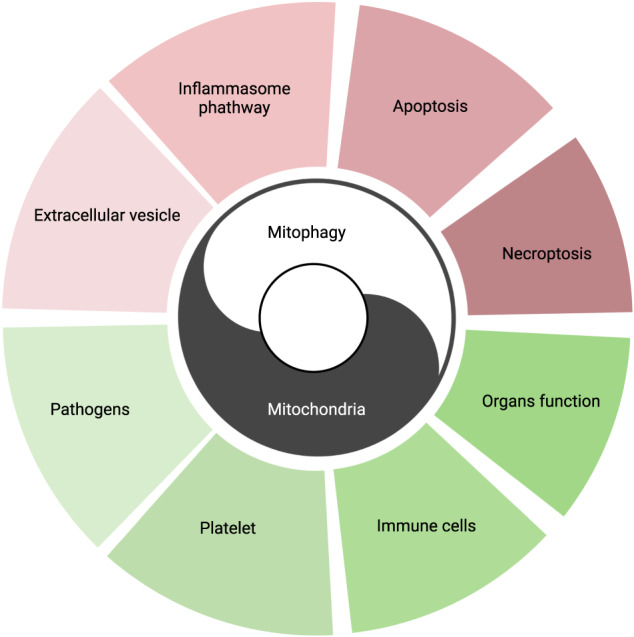
A summary of relations between mitophagy, mitochondria, and severe infection. Mitophagy and mitochondria cooperate in regulating inflammation, apoptosis, and necroptosis in the cells and extracellular vesicles. Both of them participate in regulating multiple pathophysiological processes in severe infection, such as organ functions, platelet and immune cell functions, and eliminating pathogens.

In conclusion, mitochondria with mitophagy have complicated roles in regulating the functions of cells and organs (Fig. 4). Promoting mitophagy at proper time helps to prevent and treat severe infections. But before its clinical application, there are many issues to be addressed.

### Supplementary information

aj-checklist

## Data Availability

All data analyzed during this study are included in this manuscript.

## References

[1] Mehta P, McAuley DF, Brown M, Sanchez E, Tattersall RS, Manson JJ (2020). COVID-19: consider cytokine storm syndromes and immunosuppression. Lancet.

[2] Rudd KE, Johnson SC, Agesa KM, Shackelford KA, Tsoi D, Kievlan DR (2020). Global, regional, and national sepsis incidence and mortality, 1990-2017: analysis for the Global Burden of Disease Study. Lancet.

[3] Supinski GS, Schroder EA, Callahan LA (2020). Mitochondria and critical illness. Chest.

[4] Fan L, Wu D, Goremykin V, Xiao J, Xu Y, Garg S (2020). Phylogenetic analyses with systematic taxon sampling show that mitochondria branch within Alphaproteobacteria. Nat Ecol Evol.

[5] Doerrier C, García JA, Volt H, Díaz-Casado ME, Lima-Cabello E, Ortiz F (2015). Identification of mitochondrial deficits and melatonin targets in liver of septic mice by high-resolution respirometry. Life Sci.

[6] Bock FJ, Tait SWG (2020). Mitochondria as multifaceted regulators of cell death. Nat Rev Mol Cell Biol.

[7] Harrington JS, Huh JW, Schenck EJ, Nakahira K, Siempos II, Choi AMK (2019). Circulating mitochondrial DNA as predictor of mortality in critically Ill patients: a systematic review of clinical studies. Chest.

[8] Scozzi D, Cano M, Ma L, Zhou D, Zhu JH, O’Halloran JA, et al. Circulating mitochondrial DNA is an early indicator of severe illness and mortality from COVID-19. JCI Insight. 2021;6. 10.1172/jci.insight.143299 PMC7934921 33444289

[9] Lu Y, Li Z, Zhang S, Zhang T, Liu Y, Zhang L (2023). Cellular mitophagy: mechanism, roles in diseases and small molecule pharmacological regulation. Theranostics.

[10] Vargas JNS, Hamasaki M, Kawabata T, Youle RJ, Yoshimori T (2022). The mechanisms and roles of selective autophagy in mammals. Nat Rev Mol Cell Biol.

[11] Lazarou M, Sliter DA, Kane LA, Sarraf SA, Wang C, Burman JL (2015). The ubiquitin kinase PINK1 recruits autophagy receptors to induce mitophagy. Nature.

[12] Chen Y, Dorn GW (2013). PINK1-phosphorylated mitofusin 2 is a Parkin receptor for culling damaged mitochondria. Science.

[13] Buneeva O, Medvedev A. Atypical ubiquitination and Parkinson’s disease. Int J Mol Sci. 2022;23. 10.3390/ijms23073705 PMC8998352 35409068

[14] Bingol B, Tea JS, Phu L, Reichelt M, Bakalarski CE, Song Q (2014). The mitochondrial deubiquitinase USP30 opposes parkin-mediated mitophagy. Nature.

[15] Niu K, Fang H, Chen Z, Zhu Y, Tan Q, Wei D (2020). USP33 deubiquitinates PRKN/parkin and antagonizes its role in mitophagy. Autophagy.

[16] Qin X, Wang R, Xu H, Tu L, Chen H, Li H (2022). Identification of an autoinhibitory, mitophagy-inducing peptide derived from the transmembrane domain of USP30. Autophagy.

[17] Ma Q, Xin J, Peng Q, Li N, Sun S, Hou H (2023). UBQLN2 and HSP70 participate in Parkin-mediated mitophagy by facilitating outer mitochondrial membrane rupture. EMBO Rep.

[18] Tanaka A, Cleland MM, Xu S, Narendra DP, Suen DF, Karbowski M (2010). Proteasome and p97 mediate mitophagy and degradation of mitofusins induced by Parkin. J Cell Biol.

[19] McLelland GL, Goiran T, Yi W, Dorval G, Chen CX, Lauinger ND, et al. Mfn2 ubiquitination by PINK1/parkin gates the p97-dependent release of ER from mitochondria to drive mitophagy. eLife. 2018;7. 10.7554/eLife.32866 PMC5927771 29676259

[20] Chung KP, Hsu CL, Fan LC, Huang Z, Bhatia D, Chen YJ (2019). Mitofusins regulate lipid metabolism to mediate the development of lung fibrosis. Nat Commun.

[21] Xiang H, Zhang J, Lin C, Zhang L, Liu B, Ouyang L (2020). Targeting autophagy-related protein kinases for potential therapeutic purpose. Acta Pharm Sin B.

[22] Vargas JNS, Wang C, Bunker E, Hao L, Maric D, Schiavo G (2019). Spatiotemporal control of ULK1 activation by NDP52 and TBK1 during selective autophagy. Mol Cell.

[23] Le Guerroué F, Bunker EN, Rosencrans WM, Nguyen JT, Basar MA, Werner A (2023). TNIP1 inhibits selective autophagy via bipartite interaction with LC3/GABARAP and TAX1BP1. Mol Cell.

[24] Zhou C, Ma K, Gao R, Mu C, Chen L, Liu Q (2017). Regulation of mATG9 trafficking by Src- and ULK1-mediated phosphorylation in basal and starvation-induced autophagy. Cell Res.

[25] Hurley JH, Young LN (2017). Mechanisms of autophagy initiation. Annu Rev Biochem.

[26] Zhou Z, Liu J, Fu T, Wu P, Peng C, Gong X (2021). Phosphorylation regulates the binding of autophagy receptors to FIP200 Claw domain for selective autophagy initiation. Nat Commun.

[27] Birgisdottir ÅB, Mouilleron S, Bhujabal Z, Wirth M, Sjøttem E, Evjen G (2019). Members of the autophagy class III phosphatidylinositol 3-kinase complex I interact with GABARAP and GABARAPL1 via LIR motifs. Autophagy.

[28] Nascimbeni AC, Giordano F, Dupont N, Grasso D, Vaccaro MI, Codogno P (2017). ER-plasma membrane contact sites contribute to autophagosome biogenesis by regulation of local PI3P synthesis. EMBO J.

[29] Nähse V, Raiborg C, Tan KW, Mørk S, Torgersen ML, Wenzel EM (2023). ATPase activity of DFCP1 controls selective autophagy. Nat Commun.

[30] Dooley HC, Wilson MI, Tooze SA (2015). WIPI2B links PtdIns3P to LC3 lipidation through binding ATG16L1. Autophagy.

[31] Bunker EN, Le Guerroué F, Wang C, Strub MP, Werner A, Tjandra N (2023). Nix interacts with WIPI2 to induce mitophagy. EMBO J.

[32] Wang Y, Jasper H, Toan S, Muid D, Chang X, Zhou H. Mitophagy coordinates the mitochondrial unfolded protein response to attenuate inflammation-mediated myocardial injury. Redox Biol. 2021;45. 10.1016/j.redox.2021.102049 PMC8246635 34174558

[33] Liu R, Xu C, Zhang W, Cao Y, Ye J, Li B (2022). FUNDC1-mediated mitophagy and HIF1α activation drives pulmonary hypertension during hypoxia. Cell Death Dis.

[34] Chen M, Chen Z, Wang Y, Tan Z, Zhu C, Li Y (2016). Mitophagy receptor FUNDC1 regulates mitochondrial dynamics and mitophagy. Autophagy.

[35] Yuan Y, Zheng Y, Zhang X, Chen Y, Wu X, Wu J (2017). BNIP3L/NIX-mediated mitophagy protects against ischemic brain injury independent of PARK2. Autophagy.

[36] Tang C, Han H, Liu Z, Liu Y, Yin L, Cai J (2019). Activation of BNIP3-mediated mitophagy protects against renal ischemia-reperfusion injury. Cell Death Dis.

[37] Li M, Tripathi-Giesgen I, Schulman BA, Baumeister W, Wilfling F (2023). In situ snapshots along a mammalian selective autophagy pathway. Proc Natl Acad Sci USA.

[38] Søreng K, Munson MJ, Lamb CA, Bjørndal GT, Pankiv S, Carlsson SR, et al. SNX18 regulates ATG9A trafficking from recycling endosomes by recruiting Dynamin-2. EMBO Rep. 2018;19. 10.15252/embr.201744837 PMC5891424 29437695

[39] Molino D, Zemirli N, Codogno P, Morel E (2017). The journey of the autophagosome through mammalian cell organelles and membranes. J Mol Biol.

[40] Zhao YG, Codogno P, Zhang H (2021). Machinery, regulation and pathophysiological implications of autophagosome maturation. Nat Rev Mol cell Biol.

[41] Yamano K, Wang C, Sarraf SA, Münch C, Kikuchi R, Noda NN, et al. Endosomal Rab cycles regulate Parkin-mediated mitophagy. eLife. 2018;7. 10.7554/eLife.31326 PMC5780041 29360040

[42] Jimenez-Orgaz A, Kvainickas A, Nägele H, Denner J, Eimer S, Dengjel J (2018). Control of RAB7 activity and localization through the retromer-TBC1D5 complex enables RAB7-dependent mitophagy. EMBO J.

[43] Yan BR, Li T, Coyaud E, Laurent EMN, St-Germain J, Zhou Y (2022). C5orf51 is a component of the MON1-CCZ1 complex and controls RAB7A localization and stability during mitophagy. Autophagy.

[44] Mattera R, Park SY, De Pace R, Guardia CM, Bonifacino JS (2017). AP-4 mediates export of ATG9A from the trans-Golgi network to promote autophagosome formation. Proc Natl Acad Sci USA.

[45] Ren X, Nguyen TN, Lam WK, Buffalo CZ, Lazarou M, Yokom AL (2023). Structural basis for ATG9A recruitment to the ULK1 complex in mitophagy initiation. Sci Adv.

[46] Ravussin A, Brech A, Tooze SA, Stenmark H. The phosphatidylinositol 3-phosphate-binding protein SNX4 controls ATG9A recycling and autophagy. J Cell Sci. 2021;134. 10.1242/jcs.250670 PMC7888711 33468622

[47] Yamano K, Kikuchi R, Kojima W, Hayashida R, Koyano F, Kawawaki J, et al. Critical role of mitochondrial ubiquitination and the OPTN-ATG9A axis in mitophagy. J Cell Biol. 2020;219. 10.1083/jcb.201912144 PMC7480101 32556086

[48] Shima T, Kirisako H, Nakatogawa H (2019). COPII vesicles contribute to autophagosomal membranes. J Cell Biol.

[49] Osawa T, Kotani T, Kawaoka T, Hirata E, Suzuki K, Nakatogawa H (2019). Atg2 mediates direct lipid transfer between membranes for autophagosome formation. Nat Struct Mol Biol.

[50] Maeda S, Otomo C, Otomo T. The autophagic membrane tether ATG2A transfers lipids between membranes. eLife. 2019;8. 10.7554/eLife.45777 PMC6625793 31271352

[51] Dupont N, Chauhan S, Arko-Mensah J, Castillo EF, Masedunskas A, Weigert R (2014). Neutral lipid stores and lipase PNPLA5 contribute to autophagosome biogenesis. Curr Biol.

[52] Wijdeven RH, Janssen H, Nahidiazar L, Janssen L, Jalink K, Berlin I (2016). Cholesterol and ORP1L-mediated ER contact sites control autophagosome transport and fusion with the endocytic pathway. Nat Commun.

[53] Pankiv S, Alemu EA, Brech A, Bruun JA, Lamark T, Overvatn A (2010). FYCO1 is a Rab7 effector that binds to LC3 and PI3P to mediate microtubule plus end-directed vesicle transport. J Cell Biol.

[54] Nguyen TN, Padman BS, Usher J, Oorschot V, Ramm G, Lazarou M (2016). Atg8 family LC3/GABARAP proteins are crucial for autophagosome-lysosome fusion but not autophagosome formation during PINK1/Parkin mitophagy and starvation. J Cell Biol.

[55] McEwan DG, Popovic D, Gubas A, Terawaki S, Suzuki H, Stadel D (2015). PLEKHM1 regulates autophagosome-lysosome fusion through HOPS complex and LC3/GABARAP proteins. Mol Cell.

[56] Matsui T, Jiang P, Nakano S, Sakamaki Y, Yamamoto H, Mizushima N (2018). Autophagosomal YKT6 is required for fusion with lysosomes independently of syntaxin 17. J Cell Biol.

[57] Tian X, Zheng P, Zhou C, Wang X, Ma H, Ma W (2020). DIPK2A promotes STX17- and VAMP7-mediated autophagosome-lysosome fusion by binding to VAMP7B. Autophagy.

[58] Tan HWS, Lu G, Dong H, Cho YL, Natalia A, Wang L (2022). A degradative to secretory autophagy switch mediates mitochondria clearance in the absence of the mATG8-conjugation machinery. Nat Commun.

[59] Howard M, Erickson J, Cuba Z, Kim S, Zhou W, Gade P (2022). A secretory form of Parkin-independent mitophagy contributes to the repertoire of extracellular vesicles released into the tumour interstitial fluid in vivo. J Extracell Vesicles.

[60] Towers CG, Wodetzki DK, Thorburn J, Smith KR, Caino MC, Thorburn A (2021). Mitochondrial-derived vesicles compensate for loss of LC3-mediated mitophagy. Dev Cell.

[61] Li B, Zhao H, Wu Y, Zhu Y, Zhang J, Yang G (2020). Mitochondrial-derived vesicles protect cardiomyocytes against hypoxic damage. Front Cell Dev Biol.

[62] McLelland GL, Lee SA, McBride HM, Fon EA (2016). Syntaxin-17 delivers PINK1/parkin-dependent mitochondrial vesicles to the endolysosomal system. J Cell Biol.

[63] Matheoud D, Sugiura A, Bellemare-Pelletier A, Laplante A, Rondeau C, Chemali M (2016). Parkinson’s disease-related proteins PINK1 and Parkin repress mitochondrial antigen presentation. Cell.

[64] Vasam G, Nadeau R, Cadete VJJ, Lavallée-Adam M, Menzies KJ, Burelle Y (2021). Proteomics characterization of mitochondrial-derived vesicles under oxidative stress. FASEB J.

[65] Sen A, Kallabis S, Gaedke F, Jüngst C, Boix J, Nüchel J (2022). Mitochondrial membrane proteins and VPS35 orchestrate selective removal of mtDNA. Nat Commun.

[66] Abudu YP, Shrestha BK, Zhang W, Palara A, Brenne HB, Larsen KB, et al. SAMM50 acts with p62 in piecemeal basal- and OXPHOS-induced mitophagy of SAM and MICOS components. J Cell Biol. 2021;220. 10.1083/jcb.202009092 PMC8160579 34037656

[67] Lin CW, Lo S, Perng DS, Wu DB, Lee PH, Chang YF (2014). Complete activation of autophagic process attenuates liver injury and improves survival in septic mice. Shock.

[68] Chen S, Ma J, Yin P, Liang F (2023). The landscape of mitophagy in sepsis reveals PHB1 as an NLRP3 inflammasome inhibitor. Front Immunol.

[69] Barbati C, Celia AI, Colasanti T, Vomero M, Speziali M, Putro E (2022). Autophagy hijacking in PBMC from COVID-19 patients results in lymphopenia. Front Immunol.

[70] Cai C, Tang YD, Xu G, Zheng C (2021). The crosstalk between viral RNA- and DNA-sensing mechanisms. Cell Mol Life Sci.

[71] Hou J, Han L, Zhao Z, Liu H, Zhang L, Ma C (2021). USP18 positively regulates innate antiviral immunity by promoting K63-linked polyubiquitination of MAVS. Nat Commun.

[72] Wang R, Zhu Y, Ren C, Yang S, Tian S, Chen H (2021). Influenza A virus protein PB1-F2 impairs innate immunity by inducing mitophagy. Autophagy.

[73] Ding B, Zhang L, Li Z, Zhong Y, Tang Q, Qin Y (2017). The matrix protein of human parainfluenza virus type 3 induces mitophagy that suppresses interferon responses. Cell Host Microbe.

[74] Wang K, Ma H, Liu H, Ye W, Li Z, Cheng L (2019). The glycoprotein and nucleocapsid protein of hantaviruses manipulate autophagy flux to restrain host innate immune responses. Cell Rep.

[75] Zhang B, Xu S, Liu M, Wei Y, Wang Q, Shen W (2023). The nucleoprotein of influenza A virus inhibits the innate immune response by inducing mitophagy. Autophagy.

[76] Li X, Hou P, Ma W, Wang X, Wang H, Yu Z (2022). SARS-CoV-2 ORF10 suppresses the antiviral innate immune response by degrading MAVS through mitophagy. Cell Mol Immunol.

[77] Ding B, Zhang G, Yang X, Zhang S, Chen L, Yan Q (2014). Phosphoprotein of human parainfluenza virus type 3 blocks autophagosome-lysosome fusion to increase virus production. Cell Host Microbe.

[78] Gannagé M, Dormann D, Albrecht R, Dengjel J, Torossi T, Rämer PC (2009). Matrix protein 2 of influenza A virus blocks autophagosome fusion with lysosomes. Cell Host Microbe.

[79] Hou P, Wang X, Wang H, Wang T, Yu Z, Xu C (2023). The ORF7a protein of SARS-CoV-2 initiates autophagy and limits autophagosome-lysosome fusion via degradation of SNAP29 to promote virus replication. Autophagy.

[80] Zhang Y, Sun H, Pei R, Mao B, Zhao Z, Li H (2021). The SARS-CoV-2 protein ORF3a inhibits fusion of autophagosomes with lysosomes. Cell Discov.

[81] Sun Q, Li X, Kuang E (2023). Subversion of autophagy machinery and organelle-specific autophagy by SARS-CoV-2 and coronaviruses. Autophagy.

[82] Sin J, McIntyre L, Stotland A, Feuer R, Gottlieb RA. Coxsackievirus B escapes the infected cell in ejected mitophagosomes. J Virol. 2017;91. 10.1128/JVI.01347-17 PMC5709598 28978702

[83] Mohamud Y, Shi J, Qu J, Poon T, Xue YC, Deng H (2018). Enteroviral infection inhibits autophagic flux via disruption of the SNARE complex to enhance viral replication. Cell Rep.

[84] Xu Y, Zhou P, Cheng S, Lu Q, Nowak K, Hopp AK (2019). A bacterial effector reveals the V-ATPase-ATG16L1 axis that initiates xenophagy. Cell.

[85] Choy A, Dancourt J, Mugo B, O’Connor TJ, Isberg RR, Melia TJ (2012). The Legionella effector RavZ inhibits host autophagy through irreversible Atg8 deconjugation. Science.

[86] Venet F, Monneret G (2018). Advances in the understanding and treatment of sepsis-induced immunosuppression. Nat Rev Nephrol.

[87] van der Poll T, Shankar-Hari M, Wiersinga WJ (2021). The immunology of sepsis. Immunity.

[88] Gurshaney S, Morales-Alvarez A, Ezhakunnel K, Manalo A, Huynh TH, Abe JI (2023). Metabolic dysregulation impairs lymphocyte function during severe SARS-CoV-2 infection. Commun Biol.

[89] Guo R, Zhao G, Bai G, Chen J, Han W, Cui N (2023). Depletion of mTOR ameliorates CD(4)(+) T cell pyroptosis by promoting autophagy activity in septic mice. Int Immunopharmacol.

[90] Matsuzawa Y, Oshima S, Takahara M, Maeyashiki C, Nemoto Y, Kobayashi M (2015). TNFAIP3 promotes survival of CD4 T cells by restricting MTOR and promoting autophagy. Autophagy.

[91] Ip WKE, Hoshi N, Shouval DS, Snapper S, Medzhitov R (2017). Anti-inflammatory effect of IL-10 mediated by metabolic reprogramming of macrophages. Science.

[92] Park YJ, Dodantenna N, Kim Y, Kim TH, Lee HS, Yoo YS (2023). MARCH5-dependent NLRP3 ubiquitination is required for mitochondrial NLRP3-NEK7 complex formation and NLRP3 inflammasome activation. EMBO J.

[93] Baik SH, Ramanujan VK, Becker C, Fett S, Underhill DM, Wolf AJ (2023). Hexokinase dissociation from mitochondria promotes oligomerization of VDAC that facilitates NLRP3 inflammasome assembly and activation. Sci Immunol.

[94] Yu J, Nagasu H, Murakami T, Hoang H, Broderick L, Hoffman HM (2014). Inflammasome activation leads to Caspase-1-dependent mitochondrial damage and block of mitophagy. Proc Natl Acad Sci USA.

[95] Shi F-l, Li Q, Xu R, Yuan L-s, Chen Y, Shi Z-j, et al. Blocking reverse electron transfer-mediated mitochondrial DNA oxidation rescues cells from PANoptosis. Acta Pharm Sin. 2023. 10.1038/s41401-023-01182-8 PMC10834539 37964019

[96] Zeng X, Zhang YD, Ma RY, Chen YJ, Xiang XM, Hou DY (2022). Activated Drp1 regulates p62-mediated autophagic flux and aggravates inflammation in cerebral ischemia-reperfusion via the ROS-RIP1/RIP3-exosome axis. Mil Med Res.

[97] Rosina M, Ceci V, Turchi R, Chuan L, Borcherding N, Sciarretta F (2022). Ejection of damaged mitochondria and their removal by macrophages ensure efficient thermogenesis in brown adipose tissue. Cell Metab.

[98] Baden P, Deleidi M (2016). Mitochondrial antigen presentation: a vacuolar path to autoimmunity in Parkinson’s disease. Trends Immunol.

[99] Weindel CG, Richey LJ, Mehta AJ, Shah M, Huber BT (2017). Autophagy in dendritic cells and b cells is critical for the inflammatory state of TLR7-mediated autoimmunity. J Immunol.

[100] Sliter DA, Martinez J, Hao L, Chen X, Sun N, Fischer TD (2018). Parkin and PINK1 mitigate STING-induced inflammation. Nature.

[101] Wu KKL, Long K, Lin H, Siu PMF, Hoo RLC, Ye D (2021). The APPL1-Rab5 axis restricts NLRP3 inflammasome activation through early endosomal-dependent mitophagy in macrophages. Nat Commun.

[102] Kim MJ, Bae SH, Ryu JC, Kwon Y, Oh JH, Kwon J (2016). SESN2/sestrin2 suppresses sepsis by inducing mitophagy and inhibiting NLRP3 activation in macrophages. Autophagy.

[103] Wu D, Zhang H, Wu Q, Li F, Wang Y, Liu S (2021). Sestrin 2 protects against LPS-induced acute lung injury by inducing mitophagy in alveolar macrophages. Life Sci.

[104] Wang R, Zhu Y, Lin X, Ren C, Zhao J, Wang F (2019). Influenza M2 protein regulates MAVS-mediated signaling pathway through interacting with MAVS and increasing ROS production. Autophagy.

[105] Huang T, Pu Q, Zhou C, Lin P, Gao P, Zhang X (2020). MicroRNA-302/367 cluster impacts host antimicrobial defense via regulation of mitophagic response against *Pseudomonas aeruginosa* Infection. Front Immunol.

[106] Cheng SC, Scicluna BP, Arts RJ, Gresnigt MS, Lachmandas E, Giamarellos-Bourboulis EJ (2016). Broad defects in the energy metabolism of leukocytes underlie immunoparalysis in sepsis. Nat Immunol.

[107] Bonora M, Giorgi C, Pinton P (2022). Molecular mechanisms and consequences of mitochondrial permeability transition. Nat Rev Mol Cell Biol.

[108] Lindqvist LM, Frank D, McArthur K, Dite TA, Lazarou M, Oakhill JS (2018). Autophagy induced during apoptosis degrades mitochondria and inhibits type I interferon secretion. Cell Death Differ.

[109] Pizzuto M, Pelegrin P (2020). Cardiolipin in immune signaling and cell death. Trends Cell Biol.

[110] Shen Z, Li Y, Gasparski AN, Abeliovich H, Greenberg ML (2017). Cardiolipin regulates mitophagy through the protein kinase C pathway. J Biol Chem.

[111] Wu H, Zhao X, Hochrein SM, Eckstein M, Gubert GF, Knöpper K (2023). Mitochondrial dysfunction promotes the transition of precursor to terminally exhausted T cells through HIF-1α-mediated glycolytic reprogramming. Nat Commun.

[112] Pua HH, Dzhagalov I, Chuck M, Mizushima N, He YW (2007). A critical role for the autophagy gene Atg5 in T cell survival and proliferation. J Exp Med.

[113] Pei B, Zhao M, Miller BC, Véla JL, Bruinsma MW, Virgin HW (2015). Invariant NKT cells require autophagy to coordinate proliferation and survival signals during differentiation. J Immunol.

[114] Oami T, Watanabe E, Hatano M, Sunahara S, Fujimura L, Sakamoto A (2017). Suppression of T cell autophagy results in decreased viability and function of T cells through accelerated apoptosis in a murine sepsis model. Crit Care Med.

[115] Wu Y, Chen L, Qiu Z, Zhang X, Zhao G, Lu Z (2023). PINK1 protects against dendritic cell dysfunction during sepsis through the regulation of mitochondrial quality control. Mol Med.

[116] Zhang Y, Chen L, Luo Y, Wang K, Liu X, Xiao Z (2022). Pink1/Parkin-mediated mitophagy regulated the apoptosis of dendritic cells in sepsis. Inflammation.

[117] Martinez-Martin N, Maldonado P, Gasparrini F, Frederico B, Aggarwal S, Gaya M (2017). A switch from canonical to noncanonical autophagy shapes B cell responses. Science.

[118] O’Sullivan TE, Johnson LR, Kang HH, Sun JC (2015). BNIP3- and BNIP3L-mediated mitophagy promotes the generation of natural killer cell memory. Immunity.

[119] Xu X, Araki K, Li S, Han JH, Ye L, Tan WG (2014). Autophagy is essential for effector CD8(+) T cell survival and memory formation. Nat Immunol.

[120] Chen M, Hong MJ, Sun H, Wang L, Shi X, Gilbert BE (2014). Essential role for autophagy in the maintenance of immunological memory against influenza infection. Nat Med.

[121] Riley JS, Tait SW, Mitochondrial DNA (2020). in inflammation and immunity. EMBO Rep.

[122] Zhong Z, Umemura A, Sanchez-Lopez E, Liang S, Shalapour S, Wong J (2016). NF-κB restricts inflammasome activation via elimination of damaged mitochondria. Cell.

[123] Lei Y, Wen H, Yu Y, Taxman DJ, Zhang L, Widman DG (2012). The mitochondrial proteins NLRX1 and TUFM form a complex that regulates type I interferon and autophagy. Immunity.

[124] Sun X, Sun L, Zhao Y, Li Y, Lin W, Chen D (2016). MAVS maintains mitochondrial homeostasis via autophagy. Cell Discov.

[125] Zhao B, Du F, Xu P, Shu C, Sankaran B, Bell SL (2019). A conserved PLPLRT/SD motif of STING mediates the recruitment and activation of TBK1. Nature.

[126] Herhaus L (2021). TBK1 (TANK-binding kinase 1)-mediated regulation of autophagy in health and disease. Matrix Biol: J Int Soc Matrix Biol.

[127] Heo JM, Ordureau A, Paulo JA, Rinehart J, Harper JW (2015). The PINK1-PARKIN mitochondrial ubiquitylation pathway drives a program of OPTN/NDP52 recruitment and TBK1 activation to promote mitophagy. Mol Cell.

[128] Nguyen TN, Sawa-Makarska J, Khuu G, Lam WK, Adriaenssens E, Fracchiolla D (2023). Unconventional initiation of PINK1/Parkin mitophagy by Optineurin. Mol Cell.

[129] Herhaus L, Bhaskara RM, Lystad AH, Gestal-Mato U, Covarrubias-Pinto A, Bonn F (2020). TBK1-mediated phosphorylation of LC3C and GABARAP-L2 controls autophagosome shedding by ATG4 protease. EMBO Rep.

[130] Heo JM, Ordureau A, Swarup S, Paulo JA, Shen K, Sabatini DM (2018). RAB7A phosphorylation by TBK1 promotes mitophagy via the PINK-PARKIN pathway. Sci Adv.

[131] Deretic V (2021). Autophagy in inflammation, infection, and immunometabolism. Immunity.

[132] Wu W, Wang X, Sun Y, Berleth N, Deitersen J, Schlütermann D (2021). TNF-induced necroptosis initiates early autophagy events via RIPK3-dependent AMPK activation, but inhibits late autophagy. Autophagy.

[133] Suliman HB, Kraft B, Bartz R, Chen L, Welty-Wolf KE, Piantadosi CA (2017). Mitochondrial quality control in alveolar epithelial cells damaged by S. aureus pneumonia in mice. Am J Physiol Lung Cell Mol Physiol.

[134] Ambardar SR, Hightower SL, Huprikar NA, Chung KK, Singhal A, Collen JF. Post-COVID-19 pulmonary fibrosis: novel sequelae of the current pandemic. J Clin Med. 2021;10. 10.3390/jcm10112452 PMC8199255 34205928

[135] Savin IA, Zenkova MA, Sen’kova AV. Pulmonary fibrosis as a result of acute lung inflammation: molecular mechanisms, relevant in vivo models, prognostic and therapeutic approaches. Int J Mol Sci. 2022;23. 10.3390/ijms232314959 PMC9735580 36499287

[136] Tian Z, Yao N, Wang F, Ruan L (2022). Thymosin β4 suppresses LPS-induced murine lung fibrosis by attenuating oxidative injury and alleviating inflammation. Inflammation.

[137] Sun Y, Yao X, Zhang QJ, Zhu M, Liu ZP, Ci B (2018). Beclin-1-dependent autophagy protects the heart during sepsis. Circulation.

[138] Liang S, Bao C, Yang Z, Liu S, Sun Y, Cao W (2023). SARS-CoV-2 spike protein induces IL-18-mediated cardiopulmonary inflammation via reduced mitophagy. Signal Transduct Target Ther.

[139] Pi QZ, Wang XW, Jian ZL, Chen D, Zhang C, Wu QC (2021). Melatonin alleviates cardiac dysfunction via increasing Sirt1-mediated beclin-1 deacetylation and autophagy during sepsis. Inflammation.

[140] Li L, Shi W, Zhang J, Ren L (2019). To explore the protective mechanism of PTEN-induced kinase 1 (PINK1)/Parkin mitophagy-mediated extract of periplaneta Americana on lipopolysaccharide induced cardiomyocyte injury. Med Sci Monit.

[141] Liu JX, Yang C, Zhang WH, Su HY, Liu ZJ, Pan Q (2019). Disturbance of mitochondrial dynamics and mitophagy in sepsis-induced acute kidney injury. Life Sci.

[142] Hu B-C, Zhu J-W, Wu G-H, Cai J-J, Yang X, Shao Z-Q, et al. Auto- and paracrine rewiring of NIX-mediated mitophagy by insulin-like growth factor-binding protein 7 in septic AKI escalates inflammation-coupling tubular damage. Life Sci. 2023;322. 10.1016/j.lfs.2023.121653 37011875

[143] Wang Y, Zhu J, Liu Z, Shu S, Fu Y, Liu Y (2021). The PINK1/PARK2/optineurin pathway of mitophagy is activated for protection in septic acute kidney injury. Redox Biol.

[144] Deng Z, He M, Hu H, Zhang W, Zhang Y, Ge Y, et al. Melatonin attenuates sepsis-induced acute kidney injury by promoting mitophagy through SIRT3-mediated TFAM deacetylation. Autophagy. 2023:1–15. 10.1080/15548627.2023.2252265 PMC10761103 37651673

[145] Oami T, Watanabe E, Hatano M, Teratake Y, Fujimura L, Sakamoto A (2018). Blocking liver autophagy accelerates apoptosis and mitochondrial injury in hepatocytes and reduces time to mortality in a murine sepsis model. Shock.

[146] Panzitt K, Jungwirth E, Krones E, Lee JM, Pollheimer M, Thallinger GG (2020). FXR-dependent Rubicon induction impairs autophagy in models of human cholestasis. J Hepatol.

[147] Xiong X, Ren Y, Cui Y, Li R, Wang C, Zhang Y (2017). Obeticholic acid protects mice against lipopolysaccharide-induced liver injury and inflammation. Biomed Pharmacother.

[148] Luo L, Wu J, Qiao L, Lu G, Li J, Li D (2020). Sestrin 2 attenuates sepsis-associated encephalopathy through the promotion of autophagy in hippocampal neurons. J Cell Mol Med.

[149] Qiu J, Chen Y, Zhuo J, Zhang L, Liu J, Wang B (2022). Urolithin A promotes mitophagy and suppresses NLRP3 inflammasome activation in lipopolysaccharide-induced BV2 microglial cells and MPTP-induced Parkinson’s disease model. Neuropharmacology.

[150] Ding H, Li Y, Chen S, Wen Y, Zhang S, Luo E (2022). Fisetin ameliorates cognitive impairment by activating mitophagy and suppressing neuroinflammation in rats with sepsis-associated encephalopathy. CNS Neurosci Ther.

[151] Chu C, Wang X, Yang C, Chen F, Shi L, Xu W (2023). Neutrophil extracellular traps drive intestinal microvascular endothelial ferroptosis by impairing Fundc1-dependent mitophagy. Redox Biol.

[152] Yoshihara I, Kondo Y, Okamoto K, Tanaka H. Sepsis-associated muscle wasting: a comprehensive review from bench to bedside. Int J Mol Sci. 2023;24. 10.3390/ijms24055040 PMC10003568 36902469

[153] Mankowski RT, Laitano O, Clanton TL, Brakenridge SC. Pathophysiology and treatment strategies of acute myopathy and muscle wasting after sepsis. J Clin Med. 2021;10. 10.3390/jcm10091874 PMC8123669 33926035

[154] Llano-Diez M, Fury W, Okamoto H, Bai Y, Gromada J, Larsson L (2019). RNA-sequencing reveals altered skeletal muscle contraction, E3 ligases, autophagy, apoptosis, and chaperone expression in patients with critical illness myopathy. Skelet Muscle.

[155] Leduc-Gaudet JP, Mayaki D, Reynaud O, Broering FE, Chaffer TJ, Hussain SNA, et al. Parkin overexpression attenuates sepsis-induced muscle wasting. Cells. 2020;9. 10.3390/cells9061454 PMC7349807 32545383

[156] Fuentes E, Araya-Maturana R, Urra FA (2019). Regulation of mitochondrial function as a promising target in platelet activation-related diseases. Free Radic Biol Med.

[157] Zhang W, Ma Q, Siraj S, Ney PA, Liu J, Liao X (2019). Nix-mediated mitophagy regulates platelet activation and life span. Blood Adv.

[158] Zhang W, Siraj S, Zhang R, Chen Q (2017). Mitophagy receptor FUNDC1 regulates mitochondrial homeostasis and protects the heart from I/R injury. Autophagy.

[159] Engelmann B, Massberg S (2012). Thrombosis as an intravascular effector of innate immunity. Nat Rev Immunol.

[160] Gaertner F, Massberg S (2016). Blood coagulation in immunothrombosis—at the frontline of intravascular immunity. Semin Immunol.

[161] Khismatullin RR, Ponomareva AA, Nagaswami C, Ivaeva RA, Montone KT, Weisel JW (2021). Pathology of lung-specific thrombosis and inflammation in COVID-19. J Thromb Haemost.

[162] Bonaventura A, Vecchié A, Dagna L, Martinod K, Dixon DL, Van Tassell BW (2021). Endothelial dysfunction and immunothrombosis as key pathogenic mechanisms in COVID-19. Nat Rev Immunol.

[163] Iba T, Levi M, Levy JH (2022). Intracellular communication and immunothrombosis in sepsis. J Thromb Haemost.

[164] Cao Y, Ma W, Liu Z, Pei Y, Zhu Y, Chen F (2022). Early predictive value of platelet function for clinical outcome in sepsis. J Infect.

[165] Jing H, Wu X, Xiang M, Liu L, Novakovic VA, Shi J (2022). Pathophysiological mechanisms of thrombosis in acute and long COVID-19. Front Immunol.

[166] Middleton EA, Rowley JW, Campbell RA, Grissom CK, Brown SM, Beesley SJ (2019). Sepsis alters the transcriptional and translational landscape of human and murine platelets. Blood.

[167] Edelstein CL, Venkatachalam MA, Dong Z (2020). Autophagy inhibition by chloroquine and hydroxychloroquine could adversely affect acute kidney injury and other organ injury in critically ill patients with COVID-19. Kidney Int.

[168] Tanaka T, Warner BM, Michael DG, Nakamura H, Odani T, Yin H (2022). LAMP3 inhibits autophagy and contributes to cell death by lysosomal membrane permeabilization. Autophagy.

[169] Li F, Wang Y, Song X, Wang Z, Jia J, Qing S (2022). The intestinal microbial metabolite nicotinamide N-oxide prevents herpes simplex encephalitis via activating mitophagy in microglia. Gut Microbes.

[170] Hasheminezhad SH, Boozari M, Iranshahi M, Yazarlu O, Sahebkar A, Hasanpour M (2022). A mechanistic insight into the biological activities of urolithins as gut microbial metabolites of ellagitannins. Phytother Res.

[171] Yu W, Sun S, Xu H, Li C, Ren J, Zhang Y (2020). TBC1D15/RAB7-regulated mitochondria-lysosome interaction confers cardioprotection against acute myocardial infarction-induced cardiac injury. Theranostics.

[172] Li Z, Lai M, Li J, Yang D, Zhao M, Wang D (2023). RAB7A GTPase is involved in mitophagosome formation and autophagosome-lysosome fusion in N2a cells treated with the prion protein fragment 106–126. Mol Neurobiol.

[173] Picca A, Guerra F, Calvani R, Bucci C, Lo Monaco MR, Bentivoglio AR, et al. Mitochondrial-derived vesicles as candidate biomarkers in Parkinson’s disease: rationale, design and methods of the EXosomes in PArkiNson Disease (EXPAND) study. Int J Mol Sci. 2019;20. 10.3390/ijms20102373 PMC6566801 31091653

[174] Choudhuri S, Chowdhury IH, Garg NJ (2020). Mitochondrial regulation of macrophage response against pathogens. Front Immunol.

[175] Patoli D, Mignotte F, Deckert V, Dusuel A, Dumont A, Rieu A (2020). Inhibition of mitophagy drives macrophage activation and antibacterial defense during sepsis. J Clin Investig.

[176] Lee J, Lee SA, Son SH, Choi JA, Nguyen TD, Kim J (2023). Impaired mitophagy induces antimicrobial responses in macrophages infected with Mycobacterium tuberculosis. Cell Biosci.

[177] De Snoo ML, Friesen EL, Zhang YT, Earnshaw R, Dorval G, Kapadia M (2019). Bcl-2-associated athanogene 5 (BAG5) regulates Parkin-dependent mitophagy and cell death. Cell Death Dis.

[178] Larson-Casey JL, Deshane JS, Ryan AJ, Thannickal VJ, Carter AB (2016). Macrophage Akt1 kinase-mediated mitophagy modulates apoptosis resistance and pulmonary fibrosis. Immunity.

[179] Liu Z, Qin G, Yang J, Wang W, Zhang W, Lu B (2023). Targeting mitochondrial degradation by chimeric autophagy-tethering compounds. Chem Sci.

[180] Novais AA, Chuffa LGdA, Zuccari DAPdC, Reiter RJ. Exosomes and melatonin: where their destinies intersect. Front Immunol. 2021;12. 10.3389/fimmu.2021.692022 PMC8226101 34177952

[181] Cen X, Chen Y, Xu X, Wu R, He F, Zhao Q (2020). Pharmacological targeting of MCL-1 promotes mitophagy and improves disease pathologies in an Alzheimer’s disease mouse model. Nat Commun.

[182] Youle RJ, van der Bliek AM (2012). Mitochondrial fission, fusion, and stress. Science.

[183] Liu L, Li Y, Wang J, Zhang D, Wu H, Li W (2021). Mitophagy receptor FUNDC1 is regulated by PGC-1α/NRF1 to fine tune mitochondrial homeostasis. EMBO Rep.

[184] Fang X, Wu H, Wei J, Miao R, Zhang Y, Tian J (2022). Research progress on the pharmacological effects of berberine targeting mitochondria. Front Endocrinol.

[185] Wang C, Yuan J, Du J (2021). Resveratrol alleviates acute lung injury through regulating PLSCR-3-mediated mitochondrial dysfunction and mitophagy in a cecal ligation and puncture model. Eur J Pharmacol.

[186] Reiter RJ, Sharma R, Simko F, Dominguez-Rodriguez A, Tesarik J, Neel RL (2022). Melatonin: highlighting its use as a potential treatment for SARS-CoV-2 infection. Cell Mol Life Sci.

[187] Schweers RL, Zhang J, Randall MS, Loyd MR, Li W, Dorsey FC (2007). NIX is required for programmed mitochondrial clearance during reticulocyte maturation. Proc Natl Acad Sci USA.

[188] Esteban-Martínez L, Sierra-Filardi E, McGreal RS, Salazar-Roa M, Mariño G, Seco E (2017). Programmed mitophagy is essential for the glycolytic switch during cell differentiation. EMBO J.

[189] Vo MT, Smith BJ, Nicholas J, Choi YB (2019). Activation of NIX-mediated mitophagy by an interferon regulatory factor homologue of human herpesvirus. Nat Commun.

[190] Choe SC, Hamacher-Brady A, Brady NR (2015). Autophagy capacity and sub-mitochondrial heterogeneity shape Bnip3-induced mitophagy regulation of apoptosis. Cell Commun Signal.

[191] Zhang T, Xue L, Li L, Tang C, Wan Z, Wang R (2016). BNIP3 protein suppresses PINK1 kinase proteolytic cleavage to promote mitophagy. J Biol Chem.

[192] Kodali S, Li M, Budai MM, Chen M, Wang J (2022). Protection of quiescence and longevity of IgG memory B cells by mitochondrial autophagy. J Immunol.

[193] Meng F, Sun N, Liu D, Jia J, Xiao J, Dai H (2021). BCL2L13: physiological and pathological meanings. Cell Mol Life Sci.

[194] Murakawa T, Yamaguchi O, Hashimoto A, Hikoso S, Takeda T, Oka T (2015). Bcl-2-like protein 13 is a mammalian Atg32 homologue that mediates mitophagy and mitochondrial fragmentation. Nat Commun.

[195] Murakawa T, Okamoto K, Omiya S, Taneike M, Yamaguchi O, Otsu K (2019). A Mammalian Mitophagy Receptor, Bcl2-L-13, Recruits the ULK1 Complex to Induce Mitophagy. Cell Rep.

[196] Moyzis AG, Lally NS, Liang W, Najor RH, Gustafsson Å B. Mcl-1 Differentially regulates autophagy in response to changes in energy status and mitochondrial damage. Cells. 2022;11. 10.3390/cells11091469 PMC9102819 35563775

[197] Chen G, Han Z, Feng D, Chen Y, Chen L, Wu H (2014). A regulatory signaling loop comprising the PGAM5 phosphatase and CK2 controls receptor-mediated mitophagy. Mol Cell.

[198] Huang J, Zhu T, Rong R, You M, Ji D, Li H. FUN14 domain-containing 1-mediated mitophagy suppresses interleukin-1 beta production in macrophages. Int Immunopharmacol. 2020;88. 10.1016/j.intimp.2020.106964 33182075

[199] Pan P, Chen J, Liu X, Fan J, Zhang D, Zhao W (2021). FUNDC1 Regulates Autophagy by Inhibiting ROS-NLRP3 Signaling to Avoid Apoptosis in the Lung in a Lipopolysaccharide-Induced Mouse Model. Shock.

[200] Ponneri Babuharisankar A, Kuo CL, Chou HY, Tangeda V, Fan CC, Chen CH (2023). Mitochondrial Lon-induced mitophagy benefits hypoxic resistance via Ca(2+)-dependent FUNDC1 phosphorylation at the ER-mitochondria interface. Cell Death Dis.

[201] Strappazzon F, Nazio F, Corrado M, Cianfanelli V, Romagnoli A, Fimia GM (2015). AMBRA1 is able to induce mitophagy via LC3 binding, regardless of PARKIN and p62/SQSTM1. Cell Death Differ.

[202] Di Rita A, Peschiaroli AD, Acunzo P, Strobbe D, Hu Z, Gruber J (2018). HUWE1 E3 ligase promotes PINK1/PARKIN-independent mitophagy by regulating AMBRA1 activation via IKKα. Nat Commun.

[203] Di Rienzo M, Romagnoli A, Ciccosanti F, Refolo G, Consalvi V, Arena G (2022). AMBRA1 regulates mitophagy by interacting with ATAD3A and promoting PINK1 stability. Autophagy.

[204] Yan C, Gong L, Chen L, Xu M, Abou-Hamdan H, Tang M (2020). PHB2 (prohibitin 2) promotes PINK1-PRKN/Parkin-dependent mitophagy by the PARL-PGAM5-PINK1 axis. Autophagy.

[205] Wei Y, Chiang WC, Sumpter R, Mishra P, Levine B (2017). Prohibitin 2 Is an Inner Mitochondrial Membrane Mitophagy Receptor. Cell.

